# Cephalometric Evaluation in Patients with Obstructive Sleep Apnea undergoing Lateral Pharyngoplasty

**DOI:** 10.1055/s-0043-1776718

**Published:** 2024-03-06

**Authors:** Stephanie Regiane Prata Ferreira Zanco, Bruno Bernardo Duarte, Aurélio Rochael Almeida, José Alexandre Mendonça

**Affiliations:** 1 Pontifícia Universidade Católica de Campinas (PUC-Campinas), Campinas, SP, Brazil; 2 Universidade Estadual de Campinas (UNICAMP), Campinas, SP, Brazil; 3 Postgraduate Program in Health Sciences, Pontifical Catholic University of Campinas, SP, Brazil

**Keywords:** craniofacial abnormalities, sleep apnea syndrome, cephalometrics

## Abstract

**Introduction** Lateral pharyngoplasty (LP) has shown promising results. Craniofacial deformity reduces the pharyngeal space, contributing to the etiopathogenesis. The analysis of craniofacial features can be performed using cephalometry.

**Objective** To verify if craniofacial deformity is associated with worse polysomnographic data in patients with obstructive sleep apnea (OSA), and to verify if the preoperative cephalometric parameters can interfere with the surgical success of the LP.

**Methods** The study included 21 patients with OSA aged between 18 and 65 years who underwent LP in a university hospital from 2015 to 2019. Polysomnography was performed postoperatively, after a minimum period of 6 months from the surgical procedure. In addition, a cephalometric evaluation was performed to assess craniofacial deformity, and to correlate it with the polysomnographic results.

**Results** The mean and median of all polysomnographic respiratory parameters improved postoperatively, especially the apnea-hypopnea index (AHI), which went from 40.15 to 16.60 events per hour ( *p* = 0.001). Of the 21 patients, 15 showed improvements in the AHI postoperatively. As for the cephalometric evaluations, we found that the longer the distance between the hyoid bone and the mandibular plane, the greater the patient's preoperative AHI ( *p* = 0.011). When assessing whether cephalometric changes related to craniofacial deformities influence the surgical outcome of LP, no correlation was found for any cephalometric measurement.

**Conclusion** The longer the distance between the hyoid bone and the mandibular plane, the greater the preoperative AHI, and LP was an effective OSA treatment. Cephalometric variables are not able to modify or determine the success of LP in apneic patients in the population assessed.

## Introduction

Obstructive sleep apnea (OSA) is characterized by recurrent upper airway collapse during sleep, resulting in reduced or interrupted airflow to the lungs. These pharyngeal interruptions during sleep can lead to hypercapnia, hypoxemia, and electroencephalographic arousals. 1 2

The syndrome is highly prevalent in the adult population. Zonato et al., 3 in a study with 1,042 patients who underwent polysomnography (PSG), found a prevalence of 32.8% of subjects with AHI ≥5 events per hour.

The diagnosis is based on clinical symptoms and sleep monitoring, especially with the use of type-I laboratory PSG, which is the gold standard to diagnose sleep disorders. 4

The pathophysiology of the disease is multifactorial and has not yet been fully understood; 3 it is known that the main pathophysiological mechanism of OSA involves anatomical alterations of the upper airways. They remain patent during wakefulness, but collapse during sleep, when their muscles relax. 5 Craniofacial deformity is an important factor for upper airway narrowing. 6

The gold-standard treatment for OSA is the use of a continuous positive airway pressure (PAP) device, which is associated with a wide range of health benefits. 7 Although it is highly effective in the treatment of OSA, its effectiveness is limited due to the patients' reduced long-term adherence to the therapy.

Several treatments have emerged as an alternative for patients who did not adapt to PAP devices. Among the treatment modalities, there are surgical ones, which can be divided into oropharyngeal and skeletal surgeries, palatal procedures, neurostimulation of the hypoglossal nerve, and tracheostomy. Among the oropharyngeal surgeries, uvulopalatopharyngoplasty is the most widespread in the world, with a rate of more than 80% of success in patients classified as Friedman 1. However, less than 10% of OSA patients present this anatomy. Therefore, other oropharyngeal surgeries have been developed beginning to focus on the lateral pharyngeal wall to achieve success in apneic patients with small palatine tonsils. The most outstanding of them is the lateral pharyngoplasty (LP) developed by Cahali 8 in 2003.

The procedure consists of the reconstruction of the lateral pharyngeal wall, and it has been developed and updated several times since the first publication 8 in 2003, until reaching the new technique in 2016, called version 6 of the LP. 9 It has yielded very favorable results, and it has been proven to be superior to the most performed pharyngeal surgery in the world until then: uvulopalatopharyngoplasty. The procedure can be performed regardless of the level and pattern of airway obstruction, and the size of the palatine tonsils does not interfere in the surgical decision; therefore, it can be indicated for a larger number of patients. 4 10 11 Other studies 12 on the efficacy of LP for the treatment of OSA have been carried out, and appear promising.

Striving for the development of individual and precision medicine, we reckon that there is a need to investigate the effectiveness of this treatment and to assess if there is a correlation between the success of LP and one of the most important anatomical components of the pathophysiology of the disease, which is the craniofacial composition and its consequent interference in the diameter of the upper airways.

The examination of craniofacial features can be performed through a clinical analysis of the facial profile and a radiological analysis (cephalometry). This is a non-invasive, low-cost, easy-to-perform and widely available tool for the assessment of the upper airway obstruction. 13 14 It is an examination capable of demonstrating the relationships involving craniofacial dimensions, skeletal assessment, soft tissue characteristics, and the upper airway structures in patients with OSA. 6

## Objective

The objectives of the present study were: to assess whether patients with worse craniofacial positioning present with worse preoperative PSG data; to evaluate the pre- and postoperative results of patients undergoing LP; and to correlate these data with craniofacial deformity using six cephalometric parameters.

## Materials and Methods

### Patients and Study Design

The present is an observational and prospective cross-sectional analysis of patients cared for at the General Otorhinolaryngology Outpatient Clinic of a University Hospital.

We selected 137 OSA participants of both genders, aged between 18 and 65 years, who underwent PL from 2010 to 2019. Clinical, pre- and postoperative PSG evaluations were carried out, and lateral teleradiography was requested to perform the cephalometry.

Out of the 137 patients, 46 had undergone exams before and after surgery or had agreed to undergo, after surgery, the exams that had not been performed. And out of these 46, 21 underwent version 6 of PL and agreed to undergo the radiographic examination. The process of selection of the sample is shown in Flowchart 1 . The participants were evaluated on the day scheduled for their return for the visit with the surgical team. The radiographs were obtained at the dental radiology center of the institution. The evaluation was blinded in the preoperative data and for the cephalometric analysis.

**Flowchart 1:**
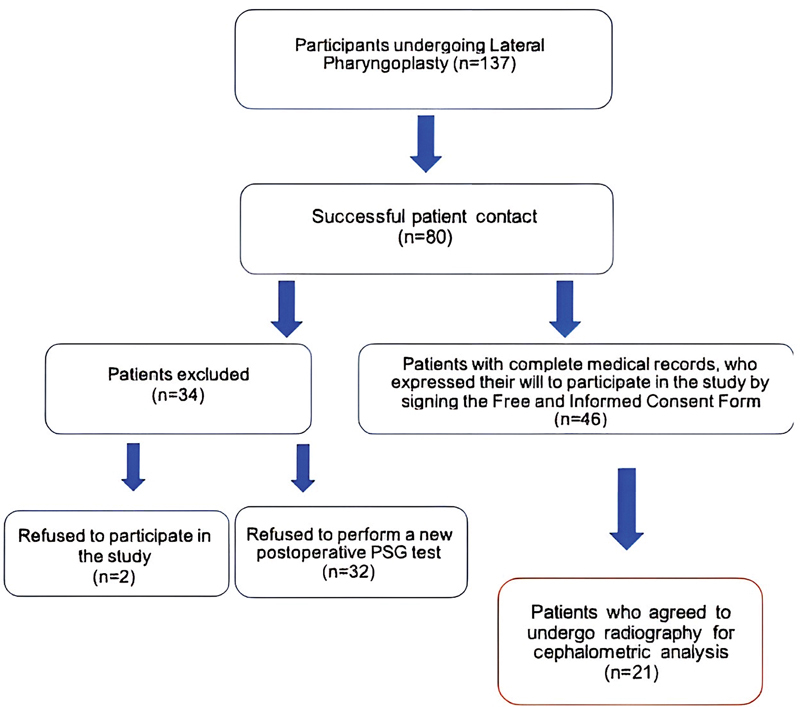
Process of selection of of participants.

The inclusion criteria were patients of both genders under evaluation at the Sleep Disorders Outpatient Clinic of the Otorhinolaryngology Department who underwent PSG before and after the surgery. The exclusion criteria were women with suspected pregnancy and those who were pregnant.

### Polysonmographic Data

The patients underwent complete type-I PSG after a minimum period of 6 months since the surgery. The evaluation of the PSG report confirming the diagnosis of OSA was performed in a specialized clinic according to the protocol recommended by Associação Brasileira do Sono (Brazilian Sleep Association), 15 and OSA was diagnosed according to the American Academy of Sleep Medicine (AASM) criteria; the examination includes the evaluation of the following parameters:

- Apnea-hypopnea index (AHI); Mild OSA: PSG – AHI ≥5 and ≤14.9, in addition to symptoms of OSA; moderate OSA: PSG – AHI > 15 and ≤ 29.9; severe OSA: PSG – AHI > 30;
- Minimum oxyhemoglobin saturation;
- Percentage of time with oxyhemoglobin saturation below 90%;
- Mean oxyhemoglobin saturation; and
- Oxygen desaturation index (ODI).

### Cephalometric Analysis

**Fig. 1:**
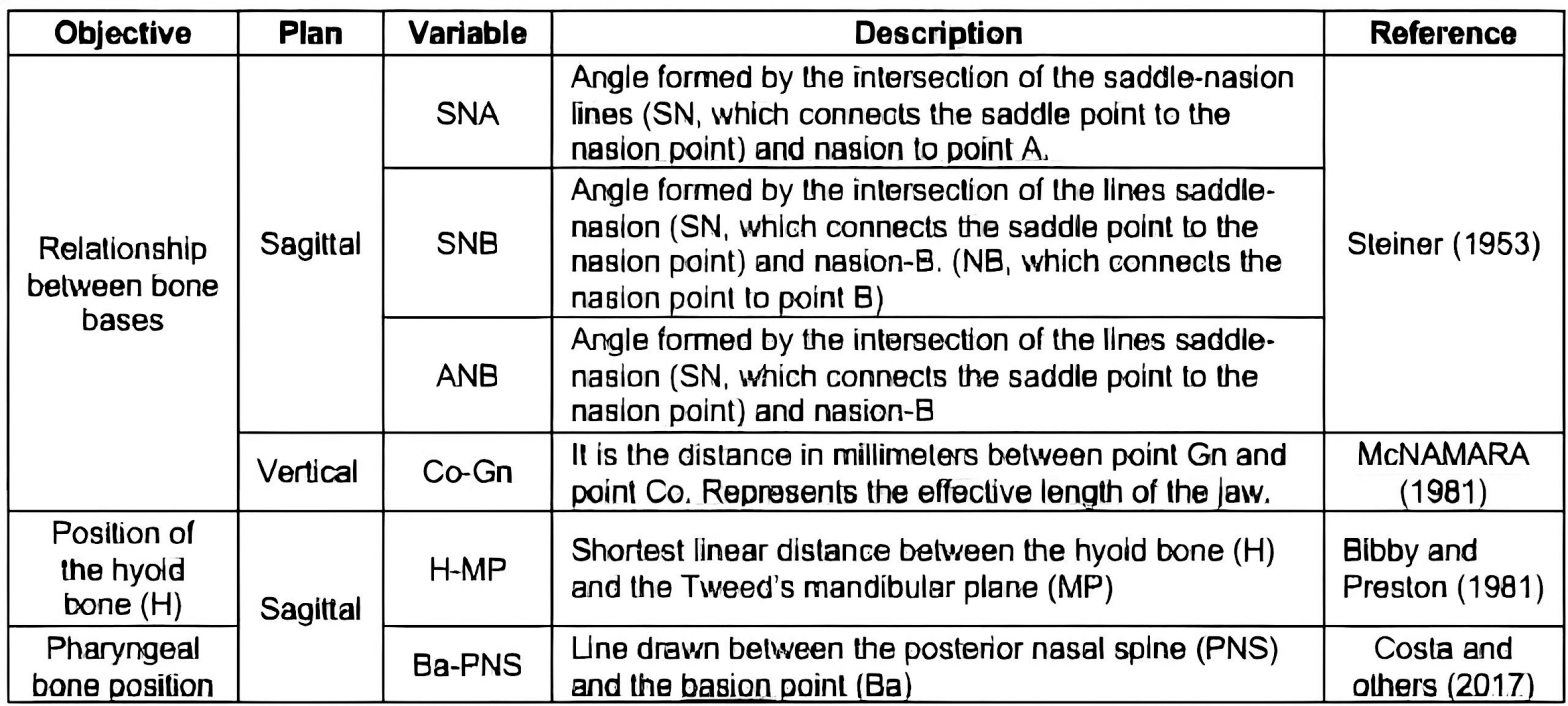
Description of variables to obtain craniofacial characteristics, sagittal and vertical cephalometric facial pattern, and measured in images generated by two-dimensional sagittal reconstruction.

We analyzed six predetermined specific craniofacial skeletal tissue cephalometric parameters, as suggested by certain studies 16 17 18 19 ( Fig. 1 ).

I. The angle formed by the intersection of sella-nasion and nasion-A lines (SNA angle): defines the degree of maxillary protrusion or retrusion in the anteroposterior direction; Reference value (RV): 82°. 16
II. The angle formed by the intersection of sella-nasion and nasion-B lines (SNB angle): demonstrates the degree of protrusion or retrusion of the mandible in the anteroposterior direction; RV: 80°. 16
III. The angle formed by the intersection of nasion-A and nasion-B lines (ANB angle): the difference between the SNA and SNB angles, representing the relationship between the maxilla and the mandible, the latter with an average value of 2°. 16
IV. Effective mandibular length (Co-Gn): the linear distance between the condyle (Co) and gnathion (Gn) points (most anterior and inferior points of the symphysis menti); RV: 120 ± 4 mm in women and 130 ± 3 mm in men. 17
V. HMP: the linear distance between the hyoid bone (H) and the mandibular plane (MP); RV: 19 mm in men and 15 mm in women. 18
VI. PNS-Ba: dimension of the bony pharynx: the linear distance between the basion and the posterior nasal spine; RV: 48 mm in men and women. 19

Lateral profile radiographs were performed at the same university center. All examinations were performed by a single operator, a radiology technician, using the a panoramic radiography equipment (Eagle Edge model, PAN/TELE configuration, serial number k000144 -Kvp 85- MA 8, Dabi Atlante, Ribeirão Preto, SP, Brazil)

Lateral profile radiographs of the face were performed with the patients in the orthostatic position, with the natural position of the head. Before exposure, all patients were instructed to bring the jaws together, keep the teeth in centric occlusion with the tip of the tongue touching the incisors, and without swallowing or talking. The radiographs were taken during the final phase of breath expiration. A cephalostat was used to hold the subject's head in a position so that the Frankfurt horizontal line was parallel to the floor during exposure.

**Fig. 2:**
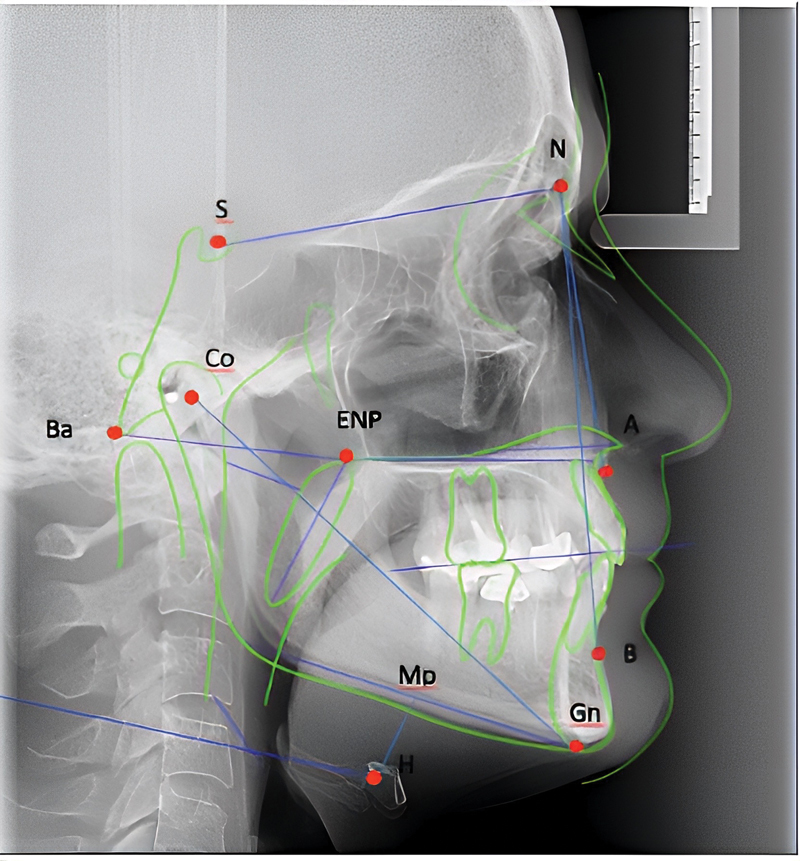
Two-dimensional sagittal reconstruction showing the cephalometric points used in the present study. Abbreviations: A: major concavity of the anterior portion of the maxilla; B: greater concavity of the anterior portion of the mandible; Ba, basion point; Co, condyle; ENP, posterior nasal spine; Gn, gnathion point; H, hyoid bone; N, nasion point (most prominent point of the frontonasal suture); S, saddle point (midpoint inside the sella turcica).

The images were grouped as digital files by the Digital Imaging and Communications in Medicine (DICOM) system and processed using the Radiocef Studio 2.0 software (Radio Memory Ltda., Belo Horizonte, MG, Brazil). The application of the software was adapted to the object of the study, measuring the recommended parameters ( Fig. 2 ). The cephalometric analyses were performed by a single investigator (oral and maxillofacial surgeon, specializing in sleep disorders). Each measurement was taken three times, and the average was used for the final review.

Based on these data, the SNA, SNB, ANB, Co-Gn, HMP, and PNS-Ba were reviewed.

### Lateral Pharyngoplasty

All patients underwent LP, and the current technique consists of the following steps:

- Place the patient in supine position under general anesthesia and orotracheal intubation and Rose position.
- Perform palatine tonsillectomy, with preservation of the posterior pillar of the tonsillar chamber (palatopharyngeal muscle, PPM)
- Remove a myomucosal triangle (palatoglossus muscle, PGM) with fat from the supratonsillar area in order to expand exposure of the lateral pharyngeal wall, when necessary.
- Detach and elevate the superior pharyngeal constrictor muscle (SPCM) from the buccopharyngeal fascia, on the posterior wall, in its cranial portion, and sectioning of it (SPCM myotomy) about 1 cm tangent to the PPM, in the region corresponding to the most discharge from the tonsillar store.
- From the inferior border of the SPCM myotomy, in a caudal direction, separate the PPM from the SPCM, creating the palatopharyngeal flap; a dissection plane is created here, since these two muscles are imbricated; this separation goes to the lower part of the tonsillar chamber.
- Reposition the flap with three Donati stitches, between the PPM and PGM, covering the SPCM, with absorbable threads on the lateral wall, leaving the repaired stitches without completing the knots yet; this suture should cover the PPM (detached from the SPCM), passing deeply through the SPCM on the lateral pharyngeal wall, up to the PGM region.
- Perform myotomy of the PPM in its caudal portion, covering the muscle and mucosa, with hemostasis of the muscle ends;
- Close the flap sutures, taking care not to strangle the tissue. These sutures are for approximation of the tissues, not to tension the structures.
- Perform a vertical relief incision between the lateral and posterior walls of the oropharynx, medially to the flap and sutures, completely separating it from the pharynx.

All steps are repeated on the opposite side, and the uvula is fully preserved. It is important that the relief incisions on the right and left sides are not too close, preserving a large island of central mucosa on the posterior wall of the oropharynx.

One of the criteria adopted to consider the success of the surgery was that of Sher et al.: 20 decrease of at least 50% in the postoperative AHI compared to the preoperative period, in addition to a final postoperative AHI < 20 events/hour.

### Statistical Analysis

Data were entered into a Microsoft Excel (Microsoft Corp., Redmond, WA, United States) spreadsheet and analyzed using descriptive and inferential analyses with the IBM SPSS Statistics for Windows (IBM Corp., Armonk, NY, United States) software, version 26.0. Initially, the continuous data were evaluated for normality distribution using the Shapiro-Wilk test; we found that the PSG variables had a non-normal distribution ( *p* < 0.05), and the cephalometric variables had a normal distribution ( *p* > 0.05). Thus, the pre-and postoperative PSG data were compared using a non-parametric test (Wilcoxon signed-rank test). After these comparisons, the calculation of the effect sizes (r coefficient) was performed for the differences found at the 5% level of significance. The distribution of the PSG variables was compared before and after surgery for each of the categories of the variable “surgical success” (Wilcoxon signed rank test) and for each of the times (pre- and postoperative periods) regarding the success or failure of the surgical procedure (Mann-Whitney U test). The pre- and postoperative parameters were also compared according to the classification of the cephalometric variables (Wilcoxon signed rank test) and, after verifying the statistically significant differences, the effect sizes were calculated at the 5% significance level. A correlation analysis was performed regarding the pre- and postoperative PSG and cephalometric variables (Spearman correlation test). Subsequently, a univariate binary logistic regression analysis was performed, and the outcome “success” regarding the surgeries was defined as follows: 1 (success), when the postoperative AHI <  20, or when the postoperative AHI < 50% of the difference between the pre- and postoperative AHI. After the univariate analyses, it was not possible to adjust a multiple explanatory model with variables significantly associated with the outcome at a significance level of 5%. For all tests, a significance level of 5% was adopted.

## Results

We evaluated 21 male patients with an average age of 39.67 (±9.93) years (minimum: 24 years; maximum: 66 years). The mean presurgical AHI (39.52 events per hour) was considered severe. Table 1 shows the characterization of the sample regarding anthropometric, PSG, and cephalometric data. By grouping several cephalometric measurements, we performed cephalometric analyses, which provide information on the sizes and shapes of craniofacial components. Therefore, the sample of the present study was composed of individuals with changes in these measurements, which means they presented craniofacial deformity.

**Table 1:** Characterization of the study sample

Variable		Mean (±standard deviation)	Median (25th percentile; 75th percentile)
**Age in years**		39.67 (±9.93)	38.00 (33.50; 46.00)
**Polysomnographic**	BMI (Kg/m ^2^ )	29.77 (±2.93)	29.39 (27.27; 32.46)
	AHI (event/hour)	39.52 (±16.95)	40.15 (24.07; 48.80)
	T < 90 (%)	22.18 (±31.99)	7.00 (0.00; 36.50)
	Minimum oxyhemoglobin saturation (%)	72.59 (±22.79)	81.00 (73.00; 85.00)
	Mean oxyhemoglobin saturation (%)	89.53 (±20.82)	94.00 (93.00; 96.00)
	ODI	52.26 (±55.96)	39.80 (16.45; 62.60)
**Anthropometric**	SNA	82.64 (±5.03)	82.24 (78.98; 86.59)
	SNB	79.84 (±4.81)	80.58 (77.17; 83.77)
	ANB	2.62 (±2.86)	2.45 (0.86; 4.53)
	HMP	18.81 (±4.95)	18.79 (15.49; 21.78)
	PNS-Ba	42.31 (±3.82)	43.09 (38.64; 44.77)
	Co-Gn	72.16 (±3.66)	71.98 (69.40; 74.53)

Abbreviations: AHI, apnea-hypopnea index; ANB, angle formed by the intersection of nasion-A and nasion-B lines; BMI, body mass index; Co-Gn, linear distance between the condyle and gnathion points; HMP, linear distance between the hyoid bone and the mandibular plane; ODI, oxygen desaturation index; PNS-Ba, linear distance between the basion and the posterior nasal spine; SNA angle formed by the intersection of sella-nasion and nasion-A lines; SNB angle formed by the intersection of sella-nasion and nasion-B lines; T < 90, percentage of time with oxyhemoglobin saturation below 90%.

Table 2 shows the comparison of pre- and postoperative PSG data. All respiratory parameters verified by PSG showed a mean improvement in the postoperative period, with an emphasis on the decrease in the AHI (from 40.15 to 16.60 events per hour), with *p* = 0.001, and an effect size considered high. With this average decrease, the surgical success described by Sher et al. 20 was achieved.

**Table 2:** Comparison between the pre- and postoperative values of the parameters evaluated

	All			
Variable	Period		*p* -value	Effect size (r coefficient)
	**Preoperative:** median (25th percentile; 75th percentile); mean (±standard deviation)	**Postoperative:** median (25th percentile; 75th percentile); mean (±standard deviation)		
** BMI (Kg/m ^2^ ) **	29.39 (27.27; 32.46) 29.77 (±2.93)	28.70 (27.41; 31.70) 29.20 (±2.47)	0.401	–
**AHI (events/hour)**	40.15 (24.07; 48.80) 39.52 (± 16.95)	16.60 (5.05; 34.75) 20.05 (± 16.08)	0.001	5.86
**T < 90 (%)**	7.00 (0.00; 36.50) 22.18 (± 31.99)	3.00 (0.00; 11.00) 11.29 (± 23.26)	0.328	–
**Minimum oxyhemoglobin** **saturation (%)**	81.00 (73.00; 85.00) 72.59 (± 22.79)	84.00 (74.00; 88.50) 78.90 (± 18.05)	0.107	–
**Mean oxyhemoglobin** **saturation (%)**	94.00 (93.00; 96.00) 89.53 (± 20.82)	95.00 (93.00; 96.00) 93.07 (± 7.37)	0.417	–
**ODI**	39.80 (16.45; 62.60) 52.26 (± 55.96)	8.80 (4.00; 30.00) 22.66 (± 33.04)	0.241	–

Abbreviations: AHI, apnea-hypopnea index; BMI, body mass index; ODI, oxygen desaturation index; T < 90, percentage of time with oxyhemoglobin saturation below 90%.

Notes: Wilcoxon signed rank test; significance level: 5%.

Table 3 shows the results of the correlation analysis regarding the anthropometric and PSG data and the cephalometric variables; we attempted to verify if there was a correlation involving worse anthropometric and polysomnographic data and worse cephalometric measurements. The preoperative body mass index (BMI) was considered moderate and positively correlated with the SNB. The preoperative AHI was shown to be moderately and positively correlated with the HMP. The Mean oxyhemoglobin saturation was moderately and positively correlated with the ANB. Postoperatively, no significant correlations were found regarding anthropometric, PSG, and cephalometric variables ( Table 3 ).

**Table 3:** Results for the correlation analysis between polysomnographic variables and cephalometric variables

	Preoperative					
Polysomnographic variables	Cephalometric variables					
	SNA	SNB	ANB	HMP	PNS-Ba	Co-Gn
** BMI (Kg/m ^2^ ) **	0.238 ( *p* = 0.457)	**0.685** **(** ***p*** ** = 0.014)**	-0.315 ( *p* = 0.319)	0.007 ( *p* = 0.983)	0.322 ( *p* = 0.308)	-0.077 ( *p* = 0.812)
**AHI (events/hour)**	-0.094 ( *p* = 0.676)	-0.047 ( *p* = 0.836)	-0.206 ( *p* = 0.357)	**0.531** **(** ***p*** ** = 0.011)**	-0.072 ( *p* = 0.751)	-0.154 ( *p* = 493)
**T < 90 (%)**	0.157 ( *p* = 0.548)	0.259 ( *p* = 0.316)	-0.358 ( *p* = 0.158)	0.356 ( *p* = 0.161)	-0.065 ( *p* = 0.805)	0.266 ( *p* = 0.302)
**Minimum oxyhemoglobin saturation (%)**	0.209 ( *p* = 0.350)	-0.061 ( *p* = 0.787)	**0.512** **(** ***p*** ** = 0.015)**	-0.292 ( *p* = 0.187)	-0.036 ( *p* = 0.875)	0.024 ( *p* = 0.914)
**Mean oxyhemoglobin** **saturation (%)**	0.409 ( *p* = 0.103)	0.300 ( *p* = 0.243)	0.339 ( *p* = 0.183)	0.269 ( *p* = 0.297)	-0.065 ( *p* = 0.805)	0.094 ( *p* = 0.718)
**ODI**	0.119 ( *p* = 0.712)	-0.007 ( *p* = 0.983)	-0.231 ( *p* = 0.471)	0.510 ( *p* = 0.090)	0.063 ( *p* = 0.846)	-0.245 ( *p* = 0.443)

Abbreviations: AHI, apnea-hypopnea index; ANB, angle formed by the intersection of nasion-A and nasion-B lines; BMI, body mass index; Co-Gn, linear distance between the condyle and gnathion points; HMP, linear distance between the hyoid bone and the mandibular plane; ODI, oxygen desaturation index; PNS-Ba, linear distance between the basion and the posterior nasal spine; SNA angle formed by the intersection of sella-nasion and nasion-A lines; SNB angle formed by the intersection of sella-nasion and nasion-B lines; T < 90, percentage of time with oxyhemoglobin saturation below 90%.

Notes: Spearman correlation test; significance level: 5%.

**Fig. 3:**
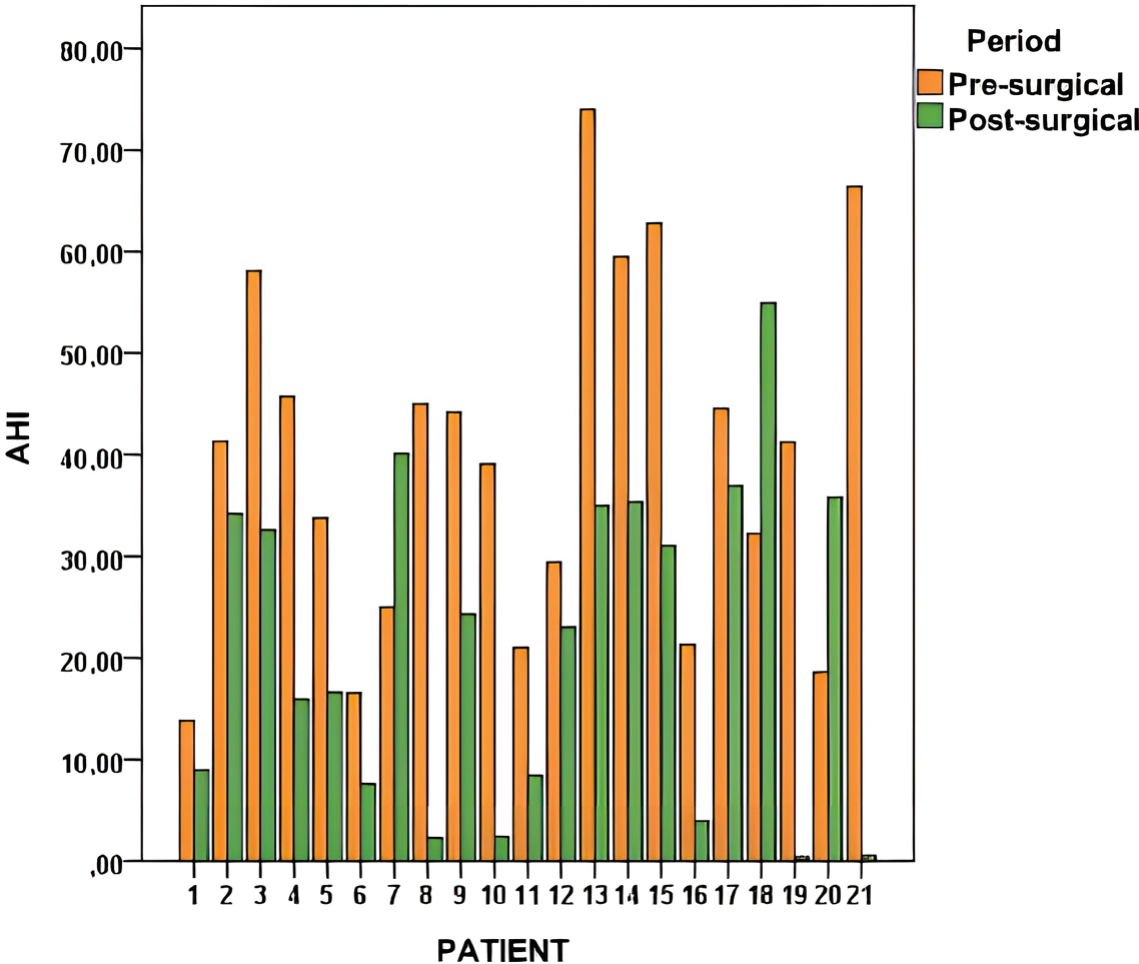
Graphic distribution of the pre- and postoperative AHI of the patients.

**Fig. 4:**
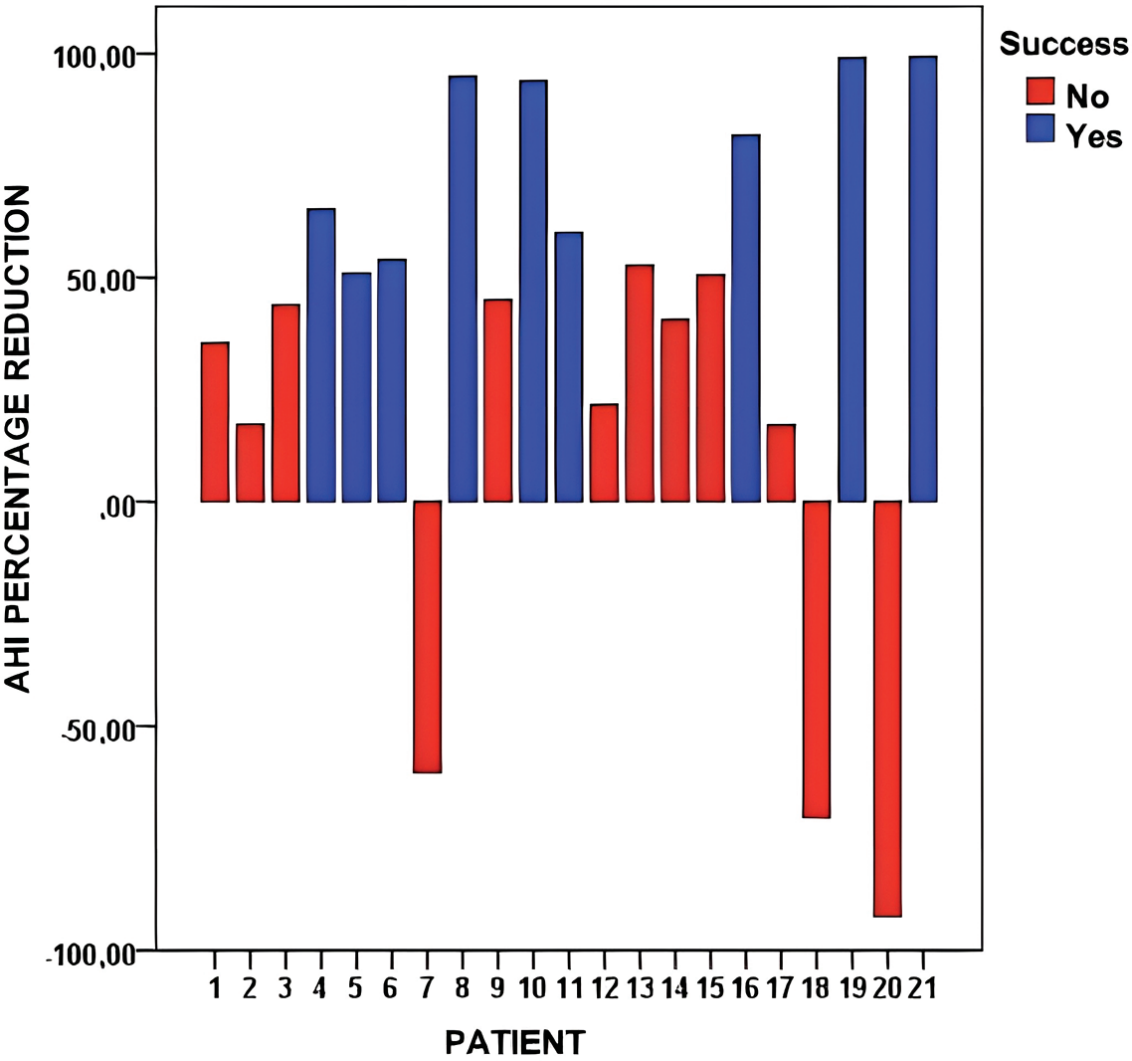
Graphic distribution of the percentage of AHI reduction for each of the patients according to surgical success defined by the Sher et al. ^20^ criteria.

Of the 21 patients who underwent LP, 17 (80.95%) presented a decrease in the AHI ( Fig. 3 ), and 9 (42.8%) achieved surgical success according to the Sher et al. 20 criteria. Based on these data, we divided the patients into two groups, those who achieved surgical success according to the Sher et al. 20 and those who did not ( Fig. 4 ). We assessed if there were any preoperative anthropometric, PSG, and cephalometric data that could predict surgical success; but these variables did not prove to be predictors, either alone (univariate analysis) or together (it was not possible to adjust a predictive multiple model of the outcome of interest) Table 4 .

**Table 4:** Evaluation of the variables of interest as predictors of the surgery success

Variable	Success		Unadjusted		Adjusted	
	No: median (25th percentile; 75th percentile)	Yes: median (25th percentile; 75th percentile)	*p*	OR (95%CI)	*p*	OR (95%CI)
**Age in years**	39.00 (34.00; 48.50)	37.00 (31.00; 46.50)	0.364	0.95 (0.87–1.05		–
** BMI (kg/m ^2^ ) **	30.80 (29.38; 32.49)	26.70 (25.40; 30.00)	0.097	0.58 (0.30–1.10)		–
**AHI (events/h)**	42.75 (26.10; 59.15)	37.58 (21.22; 45.17)	0.456	0.98 (0.93–1.03)		–
**T < 90 (%)**	8.00 (0.50; 62.00)	4.00 (0.00; 23.50)	0.542	0.36 (0.01–9.17)		–
**Minimum oxyhemoglobin** **saturation (%)**	79.00 (73.25; 82.75)	82.50 (71.25; 86.00)	0.351	10.01 (0.07–12.72)		–
**Mean oxyhemoglobin** **saturation (%)**	95.00 (93.50; 96.50)	93.50 (92.25; 95.50)	0.389	0.00 (0.00–7.25)		–
**ODI**	19.30 (6.40; 65.05)	56.45 (26.95; 99.77)	0.297	1.02 (0.98–1.06)		–
**SNA: mean (±SD)**	82.84 (±4.42)	82.39 (±5.92)	0.982	0.98 (0.82–1.16)		–
**SNB: mean (±SD)**	80.26 (±5.02)	79.34 (±4.77)	0.649	0.95 (0.80–1.14)		–
**ANB: mean (±SD)**	2.57 (±2.74)	2.67 (±2.15)	0.935	1.01 (0.75–1.36)		–
**HMP: mean (±SD)**	19.42 (±4.83)	18.08 (±4.42)	0.490	0.93 (0.77–1.13)		–
**PNS-Ba: mean (±SD)**	41.99 (±3.73)	42.69 (±4.10)	0.660	1.05 (0.83–1.32)		–
**Co-Gn: mean (±SD)**	73.37 (±3.84)	70.70 (±3.00)	0.103	0.78 (0.58–1.05)		–

Abbreviations: 95%CI, 95% confidence interval; AHI, apnea-hypopnea index; ANB, angle formed by the intersection of nasion-A and nasion-B lines; BMI, body mass index; Co-Gn, linear distance between the condyle and gnathion points; HMP, linear distance between the hyoid bone and the mandibular plane; ODI, oxygen desaturation index; OR, odds ratio; PNS-Ba, linear distance between the basion and the posterior nasal spine; SD, standard deviation; SNA angle formed by the intersection of sella-nasion and nasion-A lines; SNB angle formed by the intersection of sella-nasion and nasion-B lines; T < 90, percentage of time with oxyhemoglobin saturation below 90%.

Notes: Binary logistic regression; significance level: 5%.

## Discussion

The sample of the present study consisted of individuals with a mean age of 39.67 years, a mean BMI of 29.77 kg/m2, and a mean AHI of 39.52 events per hour of sleep. Although the surgery can be performed on obese individuals, in the present study, the sample was composed of overweight male subjects; all female patients were excluded in order to achieve a greater homogenization, but also due to the higher prevalence of OSA among male individuals. 21 22

As for the cephalometric profile, all measures were favorable to pharyngeal collapse during the night, that is, the sample was composed of individuals with craniofacial deformities.

We assessed the features of the craniofacial structures in apneic patients, seeking to associate the anatomical characteristics of these patients with the PSG parameters that represent the degree of severity of this syndrome. The SNA, SNB and ANB angles were used to verify the position of the maxilla and mandible in relation to the anterior skull base and the anteroposterior relationship between the maxilla and the mandible. The the Co-GN, HMP, and PNS-Ba. It is known that the composition of the craniofacial skeleton can contribute to airway collapsibility. 12 13 Therefore, the mean/median of these analyses were considered predisposing to upper airway collapse during sleep.

In the present study, 14 patients were identified with cephalometric variables that indicate maxillomandibular biretrusion. Research on craniofacial alterations, especially retrusions in patients with OSA, has spurred the interest of the scientific community, since this abnormality can reduce pharyngeal airflow, which can generate increased nasal resistance and impair lung functions. Furthermore, the presence of a bimaxillary retrusion, through an analysis of the angles of the skull base, can result in a reduction in the anteroposterior dimension of the pharyngeal airway in patients with OSA. 23

This piece of information becomes relevant when analyzing the surgical results of a soft tissue surgery that does not interfere with the craniofacial composition. Most patients who underwent LP presented craniofacial deformities predisposing to the development of OSA.

According to the results reported by Tepedino et al. 24 (2020) and Miles et al. 25 (1996), the mandibular length was the variable that presented a statistical correlation with the AHI. Some authors 23 26 report a positive correlation of measurements between mandibular retrognathia and the severity of OSA. Huang and Gao 6 (2020) state that the anteroposterior position of the mandible is another crucial factor, and has a notable influence on OSA, for it causes a reduction in the pharyngeal space, facilitating the collapse of the pharyngeal walls.

Studies in the literature 6 23 27 correlate cephalometric measurements with the presence and severity of OSA. The sample o the present study consisted of patients diagnosed with OSA who underwent LP. Previous studies 28 29 30 have not assessed the impact of craniofacial alterations in the success of pharyngeal surgeries; given the characteristics of the selected sample and the method adopted, the present study can be considered original.

We evaluated whether cephalometric measurements favorable to UA collapse during sleep could be correlated with worse PSG data in OSA patients. We found that the greater the HMP, the higher the AHI ( *p* = 0.011), and the greater the ANB angle, the lower the nadir oxyhemoglobin ( *p* = 0.015).

Many studies 23 27 31 32 suggest that the position of the hyoid bone plays an important role in the diagnosis and severity of OSA. This is especially due to the increase in the length of the pharynx and, consequently, its collapsible area and greater susceptibility to upper airway collapses during sleep.

In a study involving this analysis, Salles et al. 31 (2005) assessed individuals with OSA and showed that the hyoid bone is located at the level of the C4-C6 cervical vertebrae, which increases the HMP to about 27.8 mm, while in healthy individuals the average distance is of 12 mm. Kurbanova et al. 33 (2021) correlated cephalometric facial pattern measurements with the position of the hyoid bone using computed tomography (CT) in adults diagnosed with OSA, and they suggested that the hyoid bone could be a potential biological marker for OSA. In line with the present study, Ryu et al. 26 (2015) and Soares et al. 32 (2020) found a direct correlation between inferiorization of the hyoid bone and the AHI, using the HMP. Silva et al. 34 (2014) reported that this was the only variable correlated with OSA severity.

Neelapu et al. 23 (2017) reported that an increase in the ANB angle corresponds to a decrease in the oropharyngeal area. Several works are in progress to determine whether class-II malocclusion due to mandibular retrusion, with high values of the ANB angle, is a risk factor for the development of OSA, which would justify the correlation of this angle with worse oxyhemoglobin desaturations.

There are several treatment modalities for OSA, and they can be divided into non-surgical and surgical. 35 Among the surgeries, oropharyngeal and skeletal, palatal procedures, and neurostimulation of the hypoglossal nerve stand out. Oropharyngeal surgeries have shown exponential evolution in recent years, especially those involving the lateral pharyngeal wall. The most promising is LP, which was developed by Cahali 8 in 2003. Since then, there have been modifications to the technique, called versions.

In 2004, version 1 of the LP proved to be superior to the main oropharyngeal surgical technique for the treatment of OSA: uvulopalatopharyngoplasty. 10 In 2014, LP demonstrated the ability to reduce blood pressure at night and during the day in OSA patients. 12

Reports of the PSG results of version 6 of the LP are rare. According to Elsobki 35 (2021), version 6 proved to be effective when verifying that the median of the AHI decreased considerably from 41.2 to 9.5. A total of 60 percent of the patients experienced a preoperative AHI reduction greater than 50%, with a postoperative AHI < 20. Elzayat et al. 4 (2020) used version 6 in 40 patients (23 men and 17 women) submitted to PSG and sleep endoscopy before and 6 months after the procedure. Surgical success according to the Sher et al. 20 (1996) criteria was achieved in 70% of the cases, which included patients with a BMI of up to 39 Kg/m ^2^ .

In the present study, all respiratory parameters verified by PSG showed a mean postoperative improvement, with an emphasis on the reduction in the AHI (from 40.15 to 16.60 events per hour), with *p* = 0.001 and an effect size considered high. With this average reduction, surgical success according to the Sher et al. 20 (1996) criteria was achieved. Out of the 21 patients who underwent LP, 17 presented a reduction in the AHI (80.95%), and 9 (42.8%) achieved surgical success.

Currently, the criteria for the indication of LP have not been defined, neither were the anthropometric, clinical, PSG, and cephalometric data that may correlate with surgical success. Therefore, we divided the sample of the present study into two groups (patients who achieved surgical success and those who did not) to try to correlate with cephalometric data. The objective was to verify if there was any cephalometric variable that could predict the success or failure of LP.

When we evaluated the outcome of the LP (success or failure), the ratio of cephalometric measurements used did not show significant differences, evidencing that these cephalometric variables could not modify or determine the success of the surgery. Therefore, the cephalometric measurement considered unfavorable to maintaining patent UA during nightly sleep contributed to worse surgical success rates.

These data are especially relevant because they reveal the lack of need to request a cephalometric examination for the indication of LP, and they enable us to infer that craniofacial deformities may not interfere with the indication for LP in OSA patients.

The present study has certain limitations. First, the lack of a control group naturally limits the relevance of our findings. The X-ray taken with the patient in the orthostatic position and awake, not in the supine position and sleeping, in which physiological changes occur in the soft parts of the pharynx.

We can also mention the obstacles routinely encountered in clinical studies, such as the difficulties regarding patient follow-up. Exams such as CT and magnetic resonance imaging scans could not be performed due to restraints in the research budget.

In addition, we can mention a selection bias, since patients who do not have complaints and are satisfied with the surgical result in the postoperative period tend not to return to the outpatient clinic to undergo the cephalometric examination.

## Conclusion

The present study enabled us to make the following conclusions: longer MPH values presented a strong correlation with worse preoperative PSG data; LP was efficient in reducing the respiratory parameters analyzed through PSG in most of the evaluated patients; and cephalometric data did not predict the surgical outcome of LP for the treatment of OSA.
